# Gitelman Syndrome With Two Variants of Uncertain Significance: A Case Report

**DOI:** 10.7759/cureus.101667

**Published:** 2026-01-16

**Authors:** Rita Bragança, Mariana Azevedo, Ricardo Pereira, Ana Filipa Rebelo

**Affiliations:** 1 Internal Medicine, Unidade Local de Saúde de Trás-os-Montes e Alto Douro, Vila Real, PRT

**Keywords:** genetic testing, gitelman syndrome, hypokalemia, hypomagnesemia, metabolic alkalosis, salt-losing tubulopathy

## Abstract

Gitelman syndrome (GS) is an autosomal recessive salt-wasting tubulopathy caused by pathogenic variants in *SLC12A3*, characterized by renal potassium wasting, hypokalemic metabolic alkalosis, hypomagnesemia, and typically low urinary calcium excretion. We report the case of a 47-year-old woman with recurrent hypokalemia and episodes of asthenia, myalgias, and generalized muscle weakness. She denied gastrointestinal losses and diuretic or laxative use, and examination showed normal blood pressure. Electrocardiography revealed sinus rhythm without QT prolongation, and renal ultrasonography showed no structural abnormalities or nephrocalcinosis. Laboratory evaluation confirmed mild hypokalemia with metabolic alkalosis, preserved renal function, and hypomagnesemia. Urinary studies demonstrated renal potassium wasting with increased magnesium excretion and low urinary calcium excretion. Targeted next-generation sequencing identified two heterozygous *SLC12A3* missense variants of uncertain significance (VUS) (NM_000339.3:c.505G>A, p.Val169Ile; NM_000339.3:c.1452C>G, p.Cys484Trp), and segregation analysis confirmed an in-trans configuration consistent with compound heterozygosity, supporting the diagnosis in the context of a classic biochemical phenotype. She was managed with oral potassium chloride supplementation. Spironolactone was discontinued due to symptomatic hypotension, and she remains clinically stable on follow-up. This case emphasizes the value of integrating biochemical findings with segregation analysis when only VUS are identified and highlights the need for individualized long-term management and genetic counseling in GS.

## Introduction

Gitelman syndrome (GS) is an autosomal recessive salt-wasting tubulopathy caused by biallelic loss-of-function variants in the *SLC12A3* gene, which encodes the thiazide-sensitive sodium-chloride cotransporter (NCC) in the distal convoluted tubule. Impaired NCC function leads to renal sodium and chloride wasting with secondary activation of the renin-angiotensin-aldosterone system and a characteristic biochemical profile of hypokalemic metabolic alkalosis, hypomagnesemia, and low or low-normal urinary calcium excretion. GS is also referred to as familial hypokalemia-hypomagnesemia and is considered a prototypical disorder of distal convoluted tubule function [1,2].

Epidemiologic and genetic data suggest a prevalence of approximately 1 in 40,000 individuals, with a substantially higher carrier frequency and marked ethnic variation in mutation spectra, particularly in East Asian populations. Although historically regarded as a rare disease, GS is now recognized as one of the more frequent inherited renal tubular disorders [3,4].

Clinical expression is heterogeneous; some patients are diagnosed incidentally after routine blood tests showing persistent hypokalemia, whereas others present with nonspecific but often disabling manifestations such as fatigue, salt craving, thirst, nocturia, muscle weakness or cramps, paresthesias, or episodic paralysis. Despite usually preserved kidney function and normotension, health-related quality of life may be significantly reduced, and the combination of hypokalemia and hypomagnesemia can predispose to arrhythmias and neuromuscular complications [5,6].

In this context, detailed case reports remain valuable, particularly when they document the phenotype and family studies of patients carrying variants of uncertain significance (VUS). Here, we describe an adult woman with chronic hypokalemia and a classic biochemical profile of GS in whom next-generation sequencing (NGS) identified two *SLC12A3* VUS in trans, and we discuss the diagnostic, genetic, and therapeutic implications considering current evidence.

## Case presentation

A 47-year-old woman of European ancestry, with no relevant past medical history, was referred to the Internal Medicine outpatient clinic for evaluation of recurrent hypokalemia. Over the preceding 12 months, she had presented twice to the emergency department with asthenia, myalgias, and generalized muscle weakness. On both occasions, laboratory tests showed mild hypokalemia, and she was discharged on short courses of oral potassium supplementation, with transient symptomatic relief.

She denied salt craving (including a preference for salty foods or salted treats during childhood), as well as excessive thirst or abnormal drinking behavior. She reported no episodes of paresthesias or carpopedal spasms and denied a history of growth retardation or delayed puberty. In addition, she denied fainting, dizziness or vertigo, polyuria or nocturia, joint pain, palpitations, visual disturbances, or other related symptoms. She denied any gastrointestinal losses such as diarrhea or vomiting. She was not taking diuretics or laxatives and reported no recent dietary restriction. She was not taking any regular prescription medications, over-the-counter drugs, or dietary supplements. There was no history of alcohol or illicit drug use. Family history was negative for similar symptoms, electrolyte abnormalities, or kidney disease, and there was no known consanguinity.

**Figure 1:**
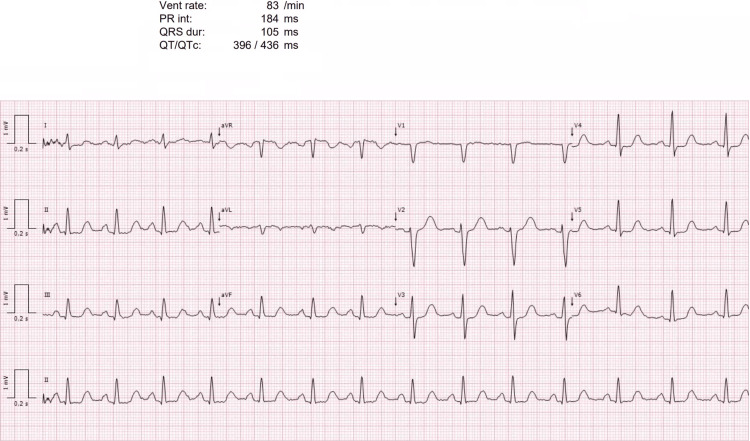
Electrocardiogram at presentation. Twelve-lead electrocardiogram showing sinus rhythm with normal PR and QRS intervals and a QTc within the reference range, with no evidence of QT prolongation.

On examination, she appeared well and euvolemic. Blood pressure was 116/74 mmHg in the seated position, and heart rate was regular. There was no peripheral edema, and neurological examination revealed full strength without focal deficits, fasciculations, or tetany. An electrocardiogram showed a normal sinus rhythm with no QT interval prolongation (Figure 1).

Baseline laboratory evaluation revealed mild hypokalemia and normal renal function. Serum sodium, chloride, total calcium, and phosphate were within reference ranges, whereas magnesium was reduced (hypomagnesemia) (Table 1).

**Table 1:** Baseline serum biochemistry at presentation.

Parameter	Result	Reference range
Urea	24	<50 mg/dL
Creatinine	0.60	0.6–1.1 mg/dL
Inorganic phosphorus	3.0	2.7–4.5 mg/dL
Total calcium	9.2	8.6–10.0 mg/dL
Magnesium	1.49	1.58–2.55 mg/dL
Sodium	140	135–147 mmol/L
Potassium	3.3	3.7–5.1 mmol/L
Chloride	101	96–106 mmol/L

An arterial blood gas confirmed metabolic alkalosis, with elevated bicarbonate (Table 2).

**Table 2:** Arterial blood gas analysis at presentation. pCO₂ = partial pressure of carbon dioxide; pO₂ = partial pressure of oxygen; HCO₃⁻ = bicarbonate

Parameter	Result	Reference range
pH (arterial)	7.47	7.35–7.45
pCO₂	44	35–45 mmHg
pO₂	94	80–100 mmHg
HCO₃⁻	30	22–26 mmol/L

Endocrine studies showed normal thyroid function and morning cortisol within the normal range. Plasma renin and aldosterone levels were compatible with secondary hyperreninemic hyperaldosteronism, as expected in a salt-wasting state. These results are summarized in Table 3.

**Table 3:** Endocrine evaluation at presentation. *: Reference for women aged >40 years. Free T4 = free thyroxine; TSH = thyroid-stimulating hormone; PTH = parathyroid hormone

Parameter	Result	Reference range
Free T4	14.1	8.24–21.0 pmol/L*
TSH	2.64	0.27–4.20 mIU/L
Morning cortisol	256.0	171–536 nmol/L
Plasma renin	49.1	2.19–38.93 pg/mL
Aldosterone	75.6	3.0–72.7 pg/mL
PTH	14.7	10.0–65.0 pg/mL

Given the chronicity of hypokalemia and the absence of obvious extrarenal losses or drug exposure, renal potassium handling was investigated. A spot potassium-to-creatinine ratio of 128 mmol/g creatinine was consistent with renal potassium wasting (>13 mmol/g creatinine) [7]. A 24-hour urine collection documented inappropriate renal potassium wasting. Urinary magnesium excretion was increased, and urinary calcium excretion was low, consistent with hypocalciuria in the context of a salt-wasting tubulopathy. Urine chloride was within the reference range (i.e., not low), which argued against vomiting or remote gastrointestinal chloride depletion as the primary mechanism (Table 4).

**Table 4:** 24-hour urine electrolytes and spot urine sample measurements at presentation.

Parameter	Result	Reference range
24-hour urine collection		
Collected volume (mL)	2,100	-
Sodium	143	85–200 mmoL/24 hours
Potassium	97	40–80 mmol/24 hours
Calcium	77	100–300 mg/24 hours
Magnesium	12	6–10 mmol/24 hours
Spot urine sample		
Calcium	6.6	6.8–21.3 mg/dL
Chloride	83	46–168 mmol/L
Potassium	57.9	20–80 mmol/L
Creatinine	45	28–217 mg/dL

**Figure 2:**
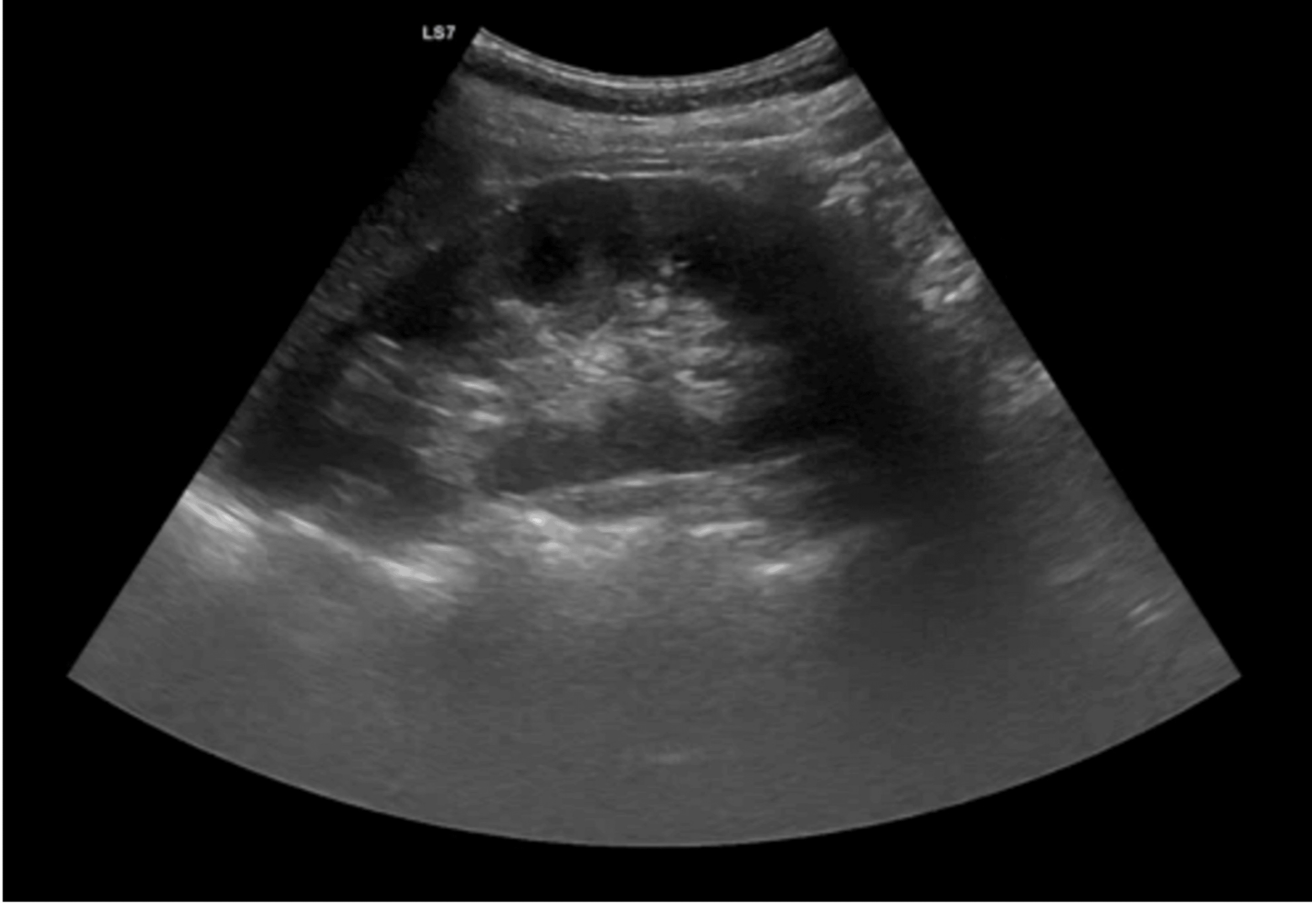
Renal ultrasonography. Long-axis ultrasound image of the kidney demonstrating preserved corticomedullary differentiation and no evidence of nephrocalcinosis or other structural abnormalities.

She also underwent renal ultrasonography, which showed no structural abnormalities and no evidence of nephrocalcinosis (Figure 2).

Based on the constellation of renal potassium wasting, metabolic alkalosis, increased magnesium excretion, and hypocalciuria, a distal convoluted tubule tubulopathy was strongly suspected. To confirm an underlying hereditary disorder, targeted NGS using an exome-based panel for Bartter syndrome and related tubulopathies (28 genes, including copy-number analysis) was performed.

Genetic testing identified two heterozygous *SLC12A3* missense VUS: NM_000339.3:c.505G>A (p.Val169Ile) in exon 3 and NM_000339.3:c.1452C>G (p.Cys484Trp) in exon 12. Both variants were reported as VUS in available databases at the time of testing. To clarify their configuration, segregation analysis was undertaken. Parental testing revealed maternal carriage of c.505G>A and paternal carriage of c.1452C>G, confirming that the variants were in trans and consistent with compound heterozygosity.

Therapeutically, the patient was started on oral potassium chloride 3,000 mg/day, with the dose titrated according to symptoms and serial electrolyte measurements. Given documented hypomagnesemia, oral magnesium supplementation was initiated with magnesium L-aspartate at 1,229.6 mg twice daily. The regimen was adjusted according to gastrointestinal tolerability and follow-up serum magnesium levels, aiming to maintain magnesium within the reference range. Spironolactone was introduced as a mineralocorticoid receptor antagonist to reduce urinary potassium wasting but was discontinued because of symptomatic hypotension and dizziness. At the one-month follow-up, she reported improvement in muscle symptoms, with persistent mild hypokalemia (3.6 mmol/L) on oral supplementation, and serum magnesium had increased from 1.49 to 2.06 mg/dL on the current regimen. Because symptoms were well controlled with potassium and magnesium supplementation, without medication-related adverse effects, and blood pressure remained normal, alternative potassium-sparing agents (eplerenone or amiloride) were not initiated at that time.

The patient was referred for genetic counseling, where the autosomal recessive inheritance pattern, carrier status of her parents, and implications for other family members were discussed.

## Discussion

This case illustrates a structured approach to chronic hypokalemia and underscores contemporary challenges in the genetic diagnosis of GS, particularly when NGS reveals VUS. In our patient, the absence of gastrointestinal symptoms or laxative/diuretic use, normal blood pressure, and normal thyroid and adrenal function argued against extrarenal or endocrine etiologies. Inappropriately high urinary potassium excretion in the setting of hypokalemia established renal potassium wasting, consistent with a salt-wasting nephropathy rather than a disorder of intake or transcellular shift [8].

The biochemical profile was highly suggestive of GS. The combination of renal potassium wasting, metabolic alkalosis, increased magnesium excretion, and hypocalciuria is characteristic of GS and helps distinguish it from Bartter syndrome, diuretic abuse, and other causes of hypokalemic metabolic alkalosis (Table 5). In Bartter syndrome, urinary calcium excretion is typically normal or increased, and age at presentation is often earlier, with polyuria, volume depletion, and nephrocalcinosis reflecting a defect in the thick ascending limb of Henle’s loop [2]. In contrast, GS usually presents later in childhood or adulthood, often with nonspecific symptoms or incidental hypokalemia, and is associated with hypocalciuria and variable hypomagnesemia due to a defect in the distal convoluted tubule [2,9]. Our patient’s biochemical constellation and clinical context were therefore highly compatible with GS.

**Table 5:** Key clinical and biochemical features distinguishing Gitelman syndrome from Bartter syndrome. Both conditions are salt-wasting tubulopathies typically associated with elevated renin and aldosterone and normal-to-low blood pressure. Gitelman syndrome is more often characterized by hypocalciuria and hypomagnesemia (with possible chondrocalcinosis), whereas Bartter syndrome more often presents earlier in life and is commonly associated with hypercalciuria and nephrocalcinosis [6].

Feature	Gitelman syndrome	Bartter syndrome
Typical blood pressure	Normal to low-normal	Normal to low-normal
Urine chloride	Usually high	Usually high
Urine calcium	Low (hypocalciuria)	Normal to high (often hypercalciuria)
Serum/Urine magnesium	Often low (renal magnesium wasting)	Normal to high-normal
Renin/Aldosterone	Elevated	Elevated
Typical presentation clues	Later onset (childhood/adulthood); cramps/fatigue; chondrocalcinosis	Often earlier onset; growth issues; nephrocalcinosis

These findings align with the current pathophysiologic understanding of GS. Loss-of-function variants in *SLC12A3* impair NCC function, reducing sodium and chloride reabsorption in the distal convoluted tubule. This increases distal sodium delivery and tubular flow, enhancing potassium and magnesium secretion under the influence of aldosterone, while chronic mild volume depletion augments proximal tubular calcium reabsorption, leading to relatively low urinary calcium excretion [10,11].

The genetic results in this case exemplify the evolving complexity of *SLC12A3* variant interpretation in the NGS era. Yet, a significant proportion of individuals with a convincing GS phenotype have only one clearly pathogenic variant or harbor missense changes classified as VUS. The reasons include limited functional data, incomplete detection of structural or deep intronic variants, and the sheer number of rare missense variants observed in population databases [12].

In our patient, the identification of two rare *SLC12A3* missense variants in trans, together with a classic GS biochemical profile and exclusion of alternative causes, provides supportive genetic evidence for GS, even though both variants remain formally classified as VUS. Similar scenarios have been described in recent GS cohorts and case reports, in which VUS were later supported as disease-causing through family segregation and functional studies [12].

Under contemporary interpretative frameworks, demonstration that each variant is inherited from a different unaffected parent in an autosomal recessive disorder, together with a highly specific phenotype and exclusion of alternative explanations, can strengthen the clinical diagnosis and guide management, while the variants remain classified as VUS at the laboratory level [12].

Clinically, GS is not a benign condition despite usually preserved renal function. Studies of adult cohorts have shown significant symptom burden, including fatigue, cramps, salt craving, and cognitive complaints, with considerable impact on health-related quality of life [11]. Additionally, recurrent or severe hypokalemia and hypomagnesemia may predispose to arrhythmias, seizures, and rhabdomyolysis, particularly in the setting of intercurrent illness or QT-prolonging medications [11].

Management of GS remains largely supportive and individualized. Current consensus and reviews emphasize oral potassium and magnesium supplementation as the therapeutic cornerstone, with dosing titrated to achieve the highest tolerated levels that ameliorate symptoms and keep serum potassium near the lower end of the normal range. However, gastrointestinal intolerance and pill burden often limit adherence. Potassium-sparing agents, such as mineralocorticoid receptor antagonists (spironolactone, eplerenone) and epithelial sodium channel blockers (amiloride), may reduce urinary potassium wasting and permit lower supplement doses, but their use must be carefully balanced against the risk of hypotension and other adverse effects, especially in normotensive patients [8]. In our case, spironolactone was poorly tolerated due to symptomatic hypotension, which is consistent with reports that blood pressure in GS is frequently low-normal, and further natriuresis can be poorly tolerated.

Genetic counseling and family studies are integral components of care in GS. Identifying biallelic *SLC12A3* variants, even when classified as VUS, has implications for relatives who may be asymptomatic carriers or have subclinical biochemical abnormalities [12]. In our patient, parental testing not only clarified the variant phase but also enabled cascade counseling and the option of targeted testing in relatives, illustrating the broader value of family-based approaches beyond immediate diagnostic confirmation.

Overall, this case underscores the value of a structured evaluation of chronic hypokalemia. The presence of two rare *SLC12A3* variants in trans in a patient with a highly concordant clinical and biochemical phenotype provides supportive evidence consistent with GS, even though both variants remain formally classified as VUS. Publishing well-phenotyped cases that include VUS is particularly valuable: it strengthens genotype-phenotype correlations, enables comparison across unrelated patients, supports future reclassification as additional evidence accumulates, and offers concrete guidance to clinicians and laboratories who encounter the same variants in routine practice. Long-term management should remain individualized to symptom burden, treatment tolerance, and patient preferences, supported by ongoing electrolyte monitoring and genetic counseling as key components of comprehensive care.

## Conclusions

GS should be considered in patients with chronic hypokalemia, metabolic alkalosis, renal potassium wasting, and no evidence of extrarenal losses, particularly when accompanied by hypomagnesemia and relative hypocalciuria. In the era of NGS, genetic confirmation is desirable but may be complicated by the frequent detection of *SLC12A3* VUS. This case of an adult woman with a typical GS biochemical profile and two *SLC12A3* VUS in trans highlights the importance of integrating clinical, biochemical, and genetic findings and underscores the diagnostic value of segregation analysis. It also reinforces that effective management of GS requires individualized electrolyte supplementation, judicious use of potassium-sparing agents, regular follow-up, and genetic counseling for the patient and their family.
